# Influence of experience on cardiovascular diving reflex in professional divers

**DOI:** 10.3389/fphys.2026.1793755

**Published:** 2026-04-27

**Authors:** Subhojit Jash, Sajan Kapil, S Kanagaraj

**Affiliations:** 1 Center for Intelligent Cyber-Physical Systems, Indian Institute of Technology Guwahati, Assam, India; 2 Department of Mechanical Engineering, Indian Institute of Technology Guwahati, Assam, India; 3 Jyoti and Bhupat Mehta School of Health Sciences and Technology, Indian Institute of Technology Guwahati, Assam, India

**Keywords:** autonomic nervous system, bradycardia, diving reflex, heart rate, SCUBA

## Abstract

**Introduction:**

The underwater environment triggers the autonomic nervous system (ANS) responses during self-contained underwater breathing apparatus (SCUBA) diving, which aims to conserve oxygen during submersion. The aim of the study is to evaluate cardiovascular responses in professional SCUBA divers by analysing heart rate dynamics across dive phases and comparing responses between novice and experienced divers in a real-world setting.

**Method:**

Twenty certified divers performed standard dives to a minimum depth of 66 feet and remained there for 5 minutes as part of the protocol. The dive was divided into six phases: rest, pre-dive, descent, bottom, ascent, and post-dive. The heart rate across different dive phases and cardiovascular reflex indices, such as heart rate drop and the minimum heart rate, is calculated. Statistical analysis was performed to comprehend the differences across phases and changes in cardiovascular reflex indices between diving experience groups.

**Results:**

The statistical analysis shows noteworthy differences in heart rate across dive phases. The notable variations were between the pre-dive and bottom phases (p<0.05) and between the descent and bottom phases(p<0.05). The study highlights the persistence of bradycardia even when depth remained constant, and a difference of 7.71 percentage points in the percentage drop in heart rate between experienced and novice divers.

**Discussion:**

Experienced divers showed a pattern of lower heart-rate responses than novice divers across selected phases of the dive. The findings point to the value of further work on diver health monitoring, training, and cardiovascular adaptation in larger samples. Experienced divers should be regularly screened for cardiovascular disease to avoid any adverse events.

## 1 Introduction

Underwater diving is a recreational and professional activity that allows individuals to explore the world below water by breathing from a self-contained underwater breathing apparatus (SCUBA). It is estimated that there are approximately 8.9–13.6 million divers who participate in dive tourism each year (Schuhbauer et al., 2025). The 7516 km coastal line of India, along with its diverse underwater experiences, such as coral reefs, attracts thousands of tourists and professional divers from around the world to explore it. It allows individuals to experience the underwater environment, which is uncommon as well as challenging in daily life.

SCUBA diving poses both physiological and psychological challenges due to the high-pressure environment (Šrámek et al., 2000; Flouris and Scott, 2009). It is well established that diving causes significant changes in the autonomic nervous system (ANS) response. In an underwater environment, the hydrostatic pressure surrounding the body balances the hydrostatic pressure within the system, causing a shift in central blood volume from the lower to the upper part of the body (Norsk et al., 1986). It increases the heart volume, stroke volume and cardiac output (Norsk, 1992; Miwa et al., 1996; Shiraishi et al., 2002). It results in bradycardia and a decrease in sympathetic activity, which in turn leads to a decrease in heart rate. It is also found that cold immersion causes changes in heart rate and heart rate variability (HRV), reflecting a shift in balance between the sympathetic nervous system (SNS) and the parasympathetic nervous system (PNS). It is more prominent due to a higher trigeminocardiac reflex and stronger vasoconstriction of the periphery. The trigeminocardiac reflex is activated by cold facial stimulation and by pressure around the nose or on the skin triggers the trigeminal nerve, which activates the vagus nerve, resulting in a sudden decrease in heart rate (Lundell et al., 2020, 2021).

Apart from the physiological effects, it is observed that psychological stress experienced during diving increases the level of stress hormones, such as cortisol and norepinephrine (Anegg et al., 2002; Chen et al., 2025). Previously, studies were conducted in a hyperbaric chamber (Lund et al., 2000) and in a pool (Schipke and Pelzer, 2001; Chouchou et al., 2009) to understand the ANS response. Recent studies have focused on the effects of cold water (Lundell et al., 2020, 2021) and warm water (33.6 °C) (Fattorini et al., 2024) on the physiology of human beings. In cold water (2-4 °C), the PNS response is strong and decreases rapidly, while the SNS response increases with an increase in heart rate. The increase in PNS and SNS creates autonomic conflict, and it is associated with the risk of arrhythmia and sudden death. The effect of breathing gas composition on the ANS is primarily caused by the partial pressure of oxygen, as it affects both the PNS and the SNS. An increase in the partial pressure of oxygen has contributed to the diving-induced bradycardia and an increase in parasympathetic tone (Noh et al., 2018). It further increased during the nitrox (40% oxygen and 60% nitrogen) diving, and the effect was more pronounced when nitrox40 was used instead of air (Zenske et al., 2020). Lafère et al. (Lafère et al., 2021) studied the differences in open-circuit breathing and closed-circuit breathing and their effects on PNS and SNS responses through HRV analysis. The increase in partial pressure of oxygen increases the high-frequency power of HRV, which suggests a chemo-reflex-induced increase in vagal activity.

Prior studies of diving physiology have employed heterogeneous designs, including extreme cold water conditions (Lundell et al., 2020, 2021), Mediterranean Sea dives with limited isolation of depth versus thermal effects (Chouchou et al., 2009), mixed warm-pool and cold quarry comparisons introducing thermal effects (Lafère et al., 2021), and simulated hyperbaric chamber exposures under controlled conditions (Chen et al., 2025). While these studies have substantially advanced understanding of autonomic responses during immersion, they differ in environmental control, breathing apparatus (closed circuit rebreathing vs open circuit), gas mixtures, and population characteristics. Chen et al. (Chen et al., 2025) have examined experience-related differences in cardiovascular response during dive stages in simulated environments. The present study extends this literature by examining phase-resolved heart-rate dynamics in real-world open-circuit diving and comparing novice and experienced divers under consistent operational conditions. The aim of the study is to evaluate the cardiovascular responses in divers by analysing the heart rate dynamics across the different phases of SCUBA diving and the difference in responses between novice and experienced divers in real-world scenarios. The cardiovascular reflex indices, as mentioned in this study, are calculated for all the divers and the interaction of phase and experience of the divers is studied using a mixed-effect framework. This helped in investigating the cardiovascular responses and adaptation between novice and experienced divers.

## 2 Materials and methods

### 2.1 Subjects and diving protocol

The study recruited twenty healthy, certified divers from Neel Diving, Cochin, Kerala, India. The cohort consisted of both novice (n=9) and experienced (n=11) divers. The certification level of the divers varied from dive master to instructor level. The term novice refers to the less experienced group relative to the more experienced divers in the sample. However, this group was not novice in an absolute sense, as all participants were certified divers, and all members of the less experienced group held Dive Master certification. For descriptive grouping, the divers were classified as novice and experienced based on their years of diving experience. This is consistent with prior work, which has used years as a standard descriptor of diver experience and reported experience strata including up to five years of diving (Gouin et al., 2025). In this study, divers with less than 5 years of diving experience are considered novices, while those with 5 or more years are considered experienced. The years of diving may not reflect the actual exposure to diving, as the frequency of diving may differ. The study has integrated self-reported logged dives as the continuous exposure measure in the mixed model. The logged dives, years-based grouping (novice and experienced) and years-continuous models are reported for sensitivity analysis. The study was reviewed and approved by the institute human ethical committee (IHEC) with reference number IITG/RnD/IHEC/2025-26/08. All divers participated voluntarily in the experiment, and informed consent was obtained from each participant. All the divers are familiar with the study site. All the dives were conducted using open-circuit SCUBA. Each diver was assessed as fit for the study, and none of them was on any medication. The demographics of the experienced and novice divers are presented in **Table 1**.

**Table 1:** The demographics of the divers.

Variables	Experienced (n=11)	Novice (n=9)	p-value
	Mean (min, max)		
Age (years)	27.81 (22-36)	22 (20-25)	0.001304^a^
Height (cm)	175.54 (170-180)	174.89 (165-182)	0.94^a^
Weight (kg)	75.63 (61-105)	69 (60-78)	0.47^a^
BMI (kg/m^2^)	24.49 (18.11-31.69)	22.51 (20.28-23.62)	0.70^a^
Years of Experience (years)	9.09 (6-14)	2.67 (2-4)	0.000161^a^
Average duration (minutes)	22.85 (19.46-25.96)	22.86 (20.3-26.11)	0.99^a^
Bottom depth (feet)	77.78 (72.83-84.64)	76.80 (74.47-79.06)	0.47^b^
Logged Dives	275.77 (110-687)	64 (53-79)	0.000192^b^
Certification	Dive master (n=6) Open water Scuba Instructor (n =5)	Dive master (n=9)	0.0141^c^

^a^ Mann–Whitney U, ^b^t-test, ^c^Fisher’s exact test.

Each diver completed a single dive to at least 66 feet in a flooded freshwater quarry. The maximum quarry depth previously explored was 105 feet. It is challenging to maintain the precise depth (66 feet) in open water diving due to buoyancy control. The actual bottom depth varied between divers, and the maximum recorded depth is 85 feet. The water temperature was approximately 28 °C, and thermal protection was not required. The experiments were conducted during the day. All divers used their own neoprene diving suits and standard open-circuit SCUBA equipment, with compressed air as the breathing gas. The divers were instructed to follow a strict protocol during the dive. Initially, the heart rate data were collected for 5 minutes before they were equipped with the diving equipment, serving as a rest phase. The next phase is the pre-dive phase, where divers float on the surface of the water with minimal movement, keeping their faces above water throughout. The heart rate was continuously recorded for 3 minutes during this period. This was followed by the start of the descent phase, during which the divers descended to a minimum depth of 66 feet. The divers were instructed to remain at that depth for 5 minutes, termed the bottom phase. They were asked to remain still and avoid any tasks in this phase. However, minor buoyancy adjustment may still occur in the open water. As the divers started to ascend, the ascent phase, a decompression stop of 3 minutes at 16 feet depth was taken for safety as per U.S. Navy Diving Manual (U.S.DepartmentoftheNavy, 2016). After the dive, data were recorded for 3 minutes during the post-dive phase. All the divers followed the protocol successfully.

### 2.2 Data acquisition

The depth and duration of the dive were recorded in a dive computer (Teric Wrist Dive computer, Shearwater Research). The heart rate was continuously recorded using a chest strap belt (POLAR H10). The Continuous monitoring of heart rate across the entire dive profile allows study of dynamic, phase-specific changes instead of relying on isolated time windows. The hydrostatic pressure, immersion, workload, and anticipatory activation vary across descent, bottom, and ascent phases, as such the autonomic responses are unlikely to be uniform throughout the dive. Continuous monitoring of heart rate helps in identification of bradycardic changes and allows assessment of experience-related adaptation. The validity of the device for measuring heart rate has been demonstrated previously (Schaffarczyk et al., 2022). Schaffarczyk et al. have validated Polar H10 against the electrocardiogram for RR intervals and heart rate during resting pre- and post-exercise. The heart ate was sampled at 1 Hz. The data was stored internally on the belt during the dive and retrieved through a mobile application, while data from the dive computer is downloaded through Shearwater applications. The dive computer and the belt are synchronised manually, and recording starts from the rest phase. For analysis, the time, heart rate and the depth variations of each diver are examined simultaneously. Occasional transient measurement spikes, where consecutive heart rates exceed 25 bpm, are identified and corrected via linear interpolation. Phase-wise mean HR was computed from the resulting cleaned signal using available samples within each phase. Across the full dataset, 43 of 32931 total heart rate samples (0.13%) exceeded the spike criteria and were corrected by linear interpolation. The phase-wise correction burden was extremely low (rest:0, Pre-dive: 0.23%, Descent: 0.41%, Bottom: 0.07%, Ascent: 0.17%, Post-dive: 0.08%). Participant-wise correction burden was similarly minimal. No exclusion threshold was prespecified because the correction burden was negligible across the dataset, and no participant or phase was excluded on data-quality grounds. The dive is divided into six distinct phases: rest, pre-dive, descent, bottom, ascent, and post-dive. The mean heart rate during the pre-dive phase is defined as the operational baseline (HR_pre_dive) as it represents the cardiovascular state immediately before submersion and provides a consistent reference point for phase-wise comparison under real-world diving conditions. Sensitivity analysis is performed with rest as the baseline. All the cardiovascular reflex indices measured from the dataset are presented in **Table 2**. The mean heart rate during the other phases (HR_rest, HR_descent, HR_bottom, HR_ascent, HR_post_dive) is compared with HR_pre_dive. The slopes of descent and ascent are calculated from phase-wise ordinary least squares regression with heart rate as the dependent variable and depth as the predictors. Depth is considered positive downward, and the slope quantifies the change in heart rate per unit increase in depth. The slope remains negative for both ascent and descent. These cardiovascular reflex indices are further compared between a group of experienced divers and novice divers to study the diving reflex in each group.

**Table 2:** Cardiovascular reflex indices and their definitions.

Cardiovascular reflex indices	Definition
HR_pre_dive	Mean heart rate during the pre-dive phase (baseline).
HR_rest	Mean heart rate during the rest phase
HR_descent	Mean heart rate during the descent phase
HR_bottom	Mean heart rate during the bottom phase
HR_ascent	Mean heart rate during the ascent phase
HR_post_dive	Mean heart rate during the post-dive phase
HR_drop	Reduction in heart rate from baseline (HR_pre_dive) to the minimum value observed since the start of descent.
% HR drop	HR_drop expressed as a percentage relative to baseline (HR_pre_dive).
MinHR	Minimum heart rate observed since the start of descent.
HR_drop_50%_descent	Reduction in heart rate from baseline (HR_pre_dive) to the heart rate at 50% of depth during descent
slope_descent	Rate of change of heart rate with depth during the descent phase.
slope_ascent	Rate of change of heart rate with depth during the ascent phase.
Time_minHR	Time from the start of descent at which MinHR occurs.
depth_minHR	Depth at which MinHR occurs (measured from the start of descent).
Time_minHR_bottom	Duration to reach MinHR during the bottom phase

### 2.3 Statistical analysis

Statistical analysis was performed to evaluate heart-rate variation across the six defined dive phases and to compare derived cardiovascular indices between novice and experienced divers. The normality of the cardiovascular indices within groups was performed using the Shapiro–Wilk test, and homogeneity of variances was assessed using Levene’s test. If both groups met normality and variance homogeneity assumptions, an independent-samples t-test was applied else Mann–Whitney U test was applied. The effect size between the groups is quantified using cohen’s d with its confidence intervals. p-values are adjusted for multiple comparisons using Benjamin–Hochberg false detection rate (FDR) and the adjusted q-values are reported. The analysis was performed using Python 3.11, and the data are presented as the mean ± standard deviation (SD) for each variable. A p-value< 0.05 was considered statistically significant.

Mean heart rate (HRmean) of different phases is modelled using a linear mixed model (LMM) for the repeated measurements within participants across different phases and the self-reported number of dives of the participants. In the study number of dives or logged dives is kept as an indicator of experience and is used in the modeling. The participants with the higher number of logged dives are more experienced. The objective was to evaluate the effect of phase-related changes and logged dives (experience) on heart rate. This is modelled as an interaction (phase * logged dives) between phases and the logged dives, with phases and the logged dives as fixed effects and divers as random intercepts. Age was included as a covariate in the model. The phases were treated as a categorical effect, and the pre-dive as the reference level. Continuous predictors such as logged dives and age are mean-centered. The logged dives are implemented in the model as the log-transformed logged dives (log_10_(logged dives+1)) to reduce the skewness in dive counts. The model is fitted in Python with a random intercept for diver, and results are reported with 95% confidence intervals, with a significant difference set at p<0.05. The model was specified as:


HRmean ∼Phases*logged dives + Age +(1|Participant)

The fixed effects include intercept, phase contrast, main effect, interaction contrast and age as a covariate. The intercept represents the mean heart rate in the pre-dive phase (heart rate at the reference phase). The phase contrast evaluates the change in heart rate between the baseline phase (pre-dive) and any of the other phases experienced by the divers at the average level of logged dives and age. The main effect of logged dives represents the slope of logged dives on heart rate in the pre-dive phase. Finally, the interaction quantifies the difference in the slope of logged dives on heart rate in a given phase compared to the pre-dive phase. The Phase contrasts are evaluated within the same LMM model using estimated marginal means (EMM) derived from the fitted model. The pairwise comparisons were conducted on EMMs, and the p-values are corrected for multiple testing using the Holm correction. Model coefficients were estimated using restricted maximum likelihood (REML). For model evaluation, the marginal and conditional R^2^, Akaike Information Criterion (AIC) and Bayesian Information Criterion (BIC) are reported. The residual errors were modelled as independent and homoscedastic distributed with constant covariance, conditional on the random effects.

## 3 Results

The mean age of the participants is 25.22 ± 5.03 years and the average BMI is 23.67 ± 3.23. The median years of experience is 6 years. Significant difference was observed in the age, years of diving and logged dives. No significant difference was observed in BMI, diving depth and diving minutes. The standard deviation of depth during the bottom phase (bottom_depth_sd) was examined between groups to explain the HR outcomes. The bottom_depth_sd for the experienced divers is 3.77 feet, and for the novice divers is 1.56 feet. The bottom_depth_sd was included as the covariate, and it did not have a significant effect on heart rate. **Supplementary Table 4** in the **Supplementary Material** presents the LMM analysis with bottom_depth_sd as a covariate. There is no significant difference in depth between the experienced and novice groups, suggesting the groups have the same level of hyperbaric exposure. Sensitivity analysis was performed with year-based grouping, years of diving as a continuous variable and logged dives, and the results are presented in the supplementary document (S) in **Supplementary Tables S1**–**S3**. The year-based grouping and logged dives showed a stable and consistent phase-dependent effect, while years of diving as a continuous variable showed a similar trend but a weaker statistical value. Therefore, logged dives along with the covariates are treated as the primary experience and used in the LMM, and the 5-year grouping was used for descriptive reporting, as reported in prior literature.

**Figure 1:**
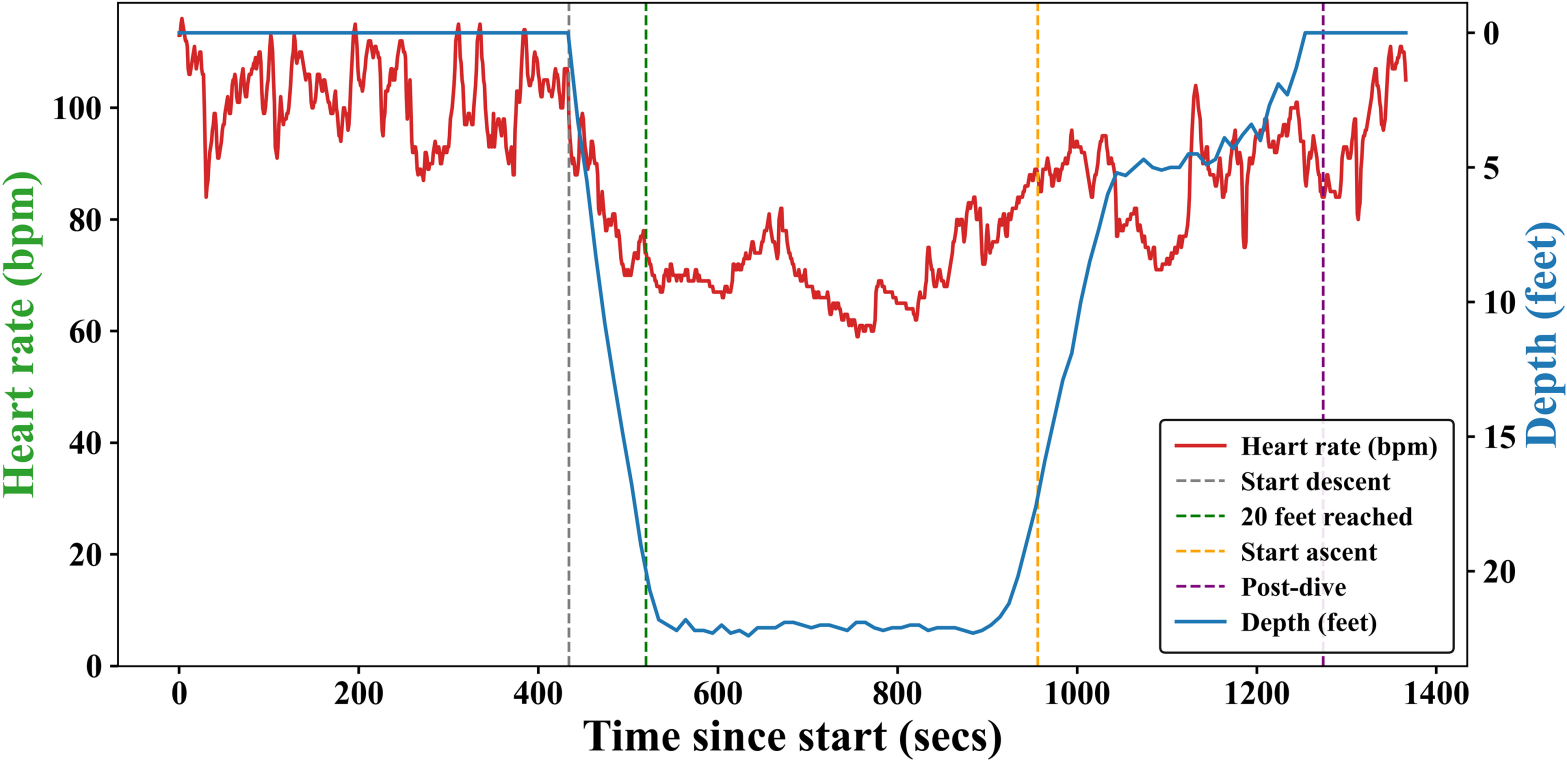
Variation of Heart rate and depth with time during diving for one of the divers.

**Figure 2:**
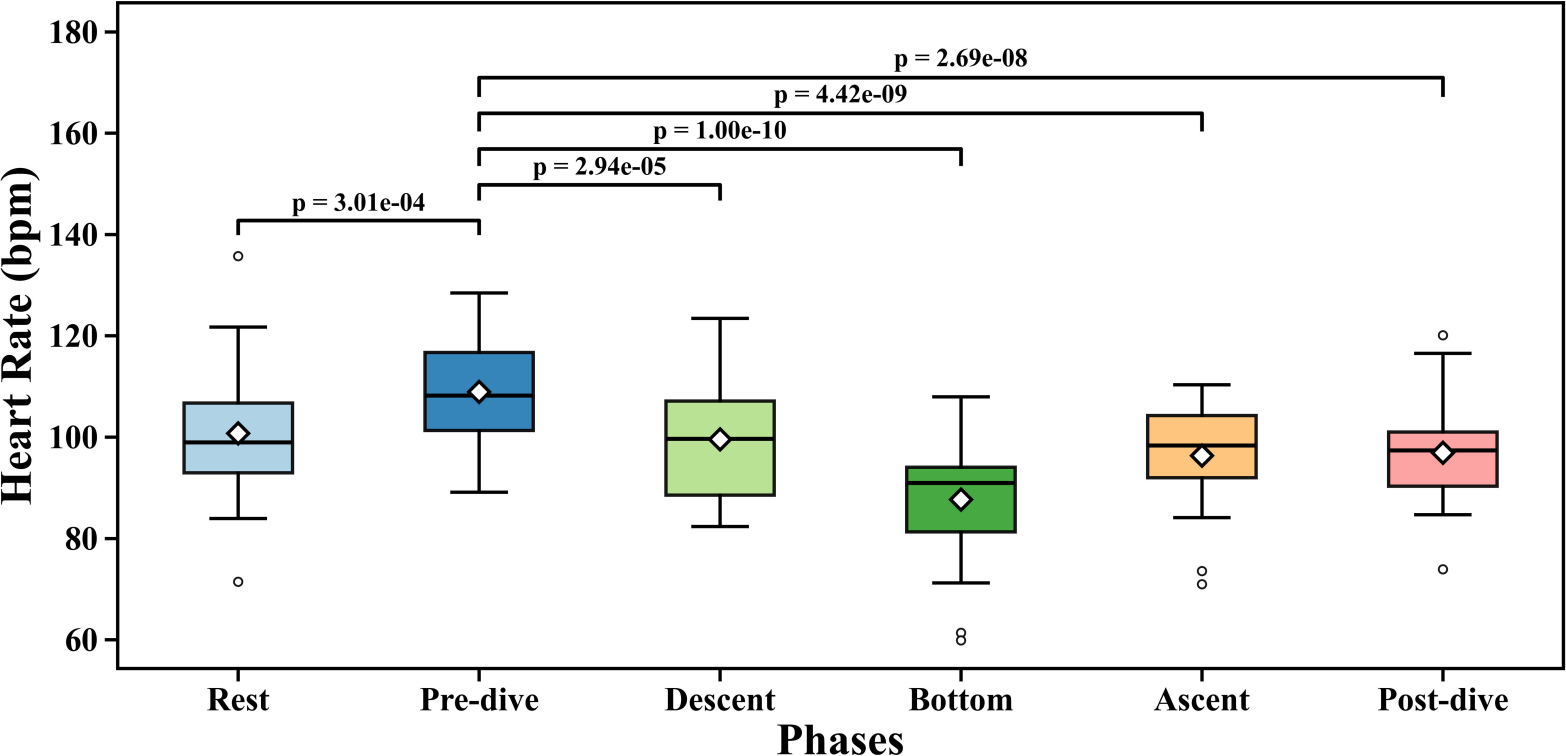
Heart rate across different phases.

Sensitivity analysis is performed with rest as the reference point to check the possibility that pre-dive HR reflects anticipatory activation. The results with rest and pre-dive as reference are given in the supplementary document (S) in **Supplementary Table 3**, **S5**. The phase*logged dives (experience) interaction effects for descent, bottom, and ascent phases remained statistically significant under both baselines. There are changes in coefficients between phases, which occur due to the re-parameterisation. During the pre-dive phase, the mean heart rate is recorded as 108.95 ± 11.30 bpm. Once the diver dived into the water, the heart rate started to decrease. The mean heart rate during the descent phase is observed to be 99.55 ± 12.23 bpm with an average descent slope of -0.305 bpm per feet. The mean of the minimum heart rate during the dive was 73.6 ± 10.20 bpm. The average depth covered during the experiment was 77.32± 2.95 feet, and the average time to reach minimum heart rate was 350.95 ± 134.20 seconds. The variation in heart rate and depth over time for one of the divers is shown in **Figure 1**. The average post-dive heart rate after resurfacing was 96.94 ± 10.9 bpm. The box plot for heart rate across different phases is shown in **Figure 2**. The heart rate drop during the dives was 35.35 ± 10.39 bpm, while the reduction from pre-dive to 50% of descent was 14.53 ± 8.52%.

**Figure 3:**
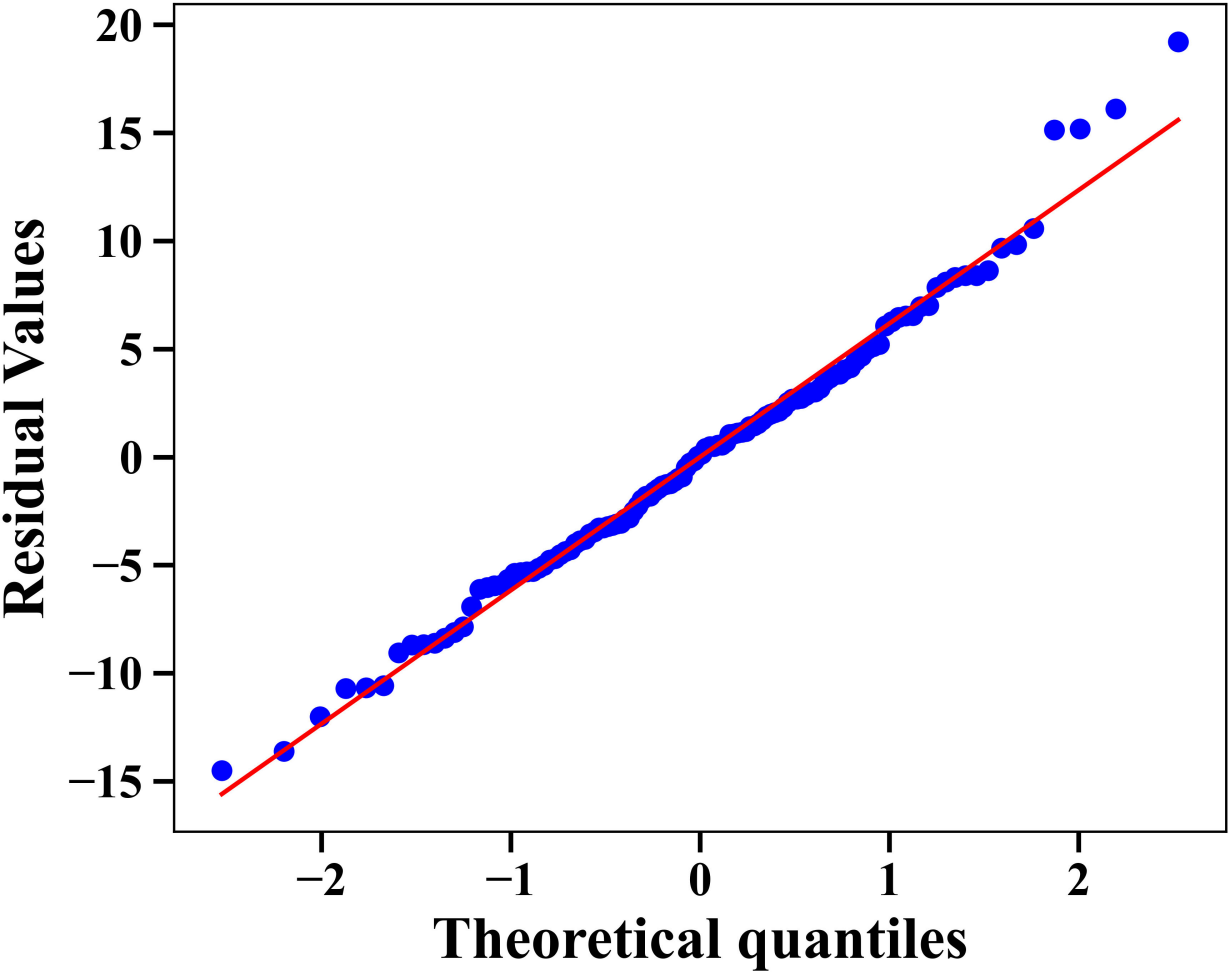
Q-Q plot for the LMM residuals.

Heart rate differed across the dive phases and depended on the logged dives of the participants. The maximum difference is observed at phase contrast, where the difference in heart rate between pre-dive and depth is 21.217 bpm. The interaction phase indicates that the relationship between logged dives and mean heart rate differs across the dive phase. In comparison to pre-dive, the change in slope was significantly more negative in descent (−14.37), depth (−15.57) and ascent (−17.43) phases. This indicates that greater logged dives (experience) is associated with lower mean heart rate in these phases. The interaction between the rest and post-dive phases is not significant. The details of the LMM model coefficients are presented in **Table 3**. The marginal R2 and the conditional R2 values are 0.321 and 0.776, respectively. The model evaluation indices from the maximum likelihood of the model are AIC = 865.53 and BIC = 907.34. The Q-Q plot is provided in **Figure 3** for the residual assumptions.

**Table 3:** LMM analysis of phase * logged dives on mean heart rate across diving stages with age as covariate.

Fixed effect	Coefficients	p-value	CI [2.5-97.5]	
Intercept	108.955	p<0.005	103.748	114.163
Main effect (Slope of logged dives on HR in Pre-dive phase)	5.273	0.724	-16.645	23.956
Phase Contrast				
Pre-dive vs. Rest	-8.218	p<0.005	-12.384	-4.053
Pre-dive vs. Descent	-9.795	p<0.005	-13.960	-5.629
Pre-dive vs. Bottom	-21.217	p<0.005	-25.382	-17.051
Pre-dive vs. Ascent	-12.649	p<0.005	-16.815	-8.484
Pre-dive vs. Post-dive	-12.012	p<0.005	-16.178	-7.847
Interaction Contrast				
Pre-dive - Rest (changes in slopes)	1.411	p=0.821	-10.842	13.665
Pre-dive – Descent (changes in slopes)	-14.367	p=0.022	-26.621	-2.114
Pre-dive – Bottom (changes in slopes)	-15.568	p=0.013	-27.821	-3.314
Pre-dive – Ascent (changes in slopes)	-17.429	p=0.005	-29.683	-5.176
Pre-dive - Post-dive (changes in slopes)	-10.261	p=0.101	-22.514	1.993
Age (Covariate)	-0.694	p=0.307	-2.026	0.638

The EMMs from the LMM show phase-related differences in mean heart rate values across the phases. HRmean for the phases are as follows: rest (EMM = 100 bpm), pre-dive (EMM = 108.93 bpm), descent (EMM = 99.56 bpm), bottom (EMM = 88.22 bpm), ascent (EMM = 96.92 bpm) and post_dive (EMM = 97.05 bpm). The *post hoc* analysis for pairwise comparison and adjusted p-values revealed significant differences in heart rate between the descent and bottom phases (p_holm = 1.69*10^-7^, mean difference =11.42 bpm), ascent and pre-dive phase (p_holm = 4.58*10^-9^, mean difference =12.64 bpm), bottom and rest phase (p_holm = 1.60*10^-9^, mean difference =12.99 bpm), pre-dive and post-dive (p_holm = 3.06*10^-8^, mean difference =12.01 bpm) and pre-dive and bottom phases (p_holm< 0.005, mean difference =21.21 bpm).

**Figure 4:**
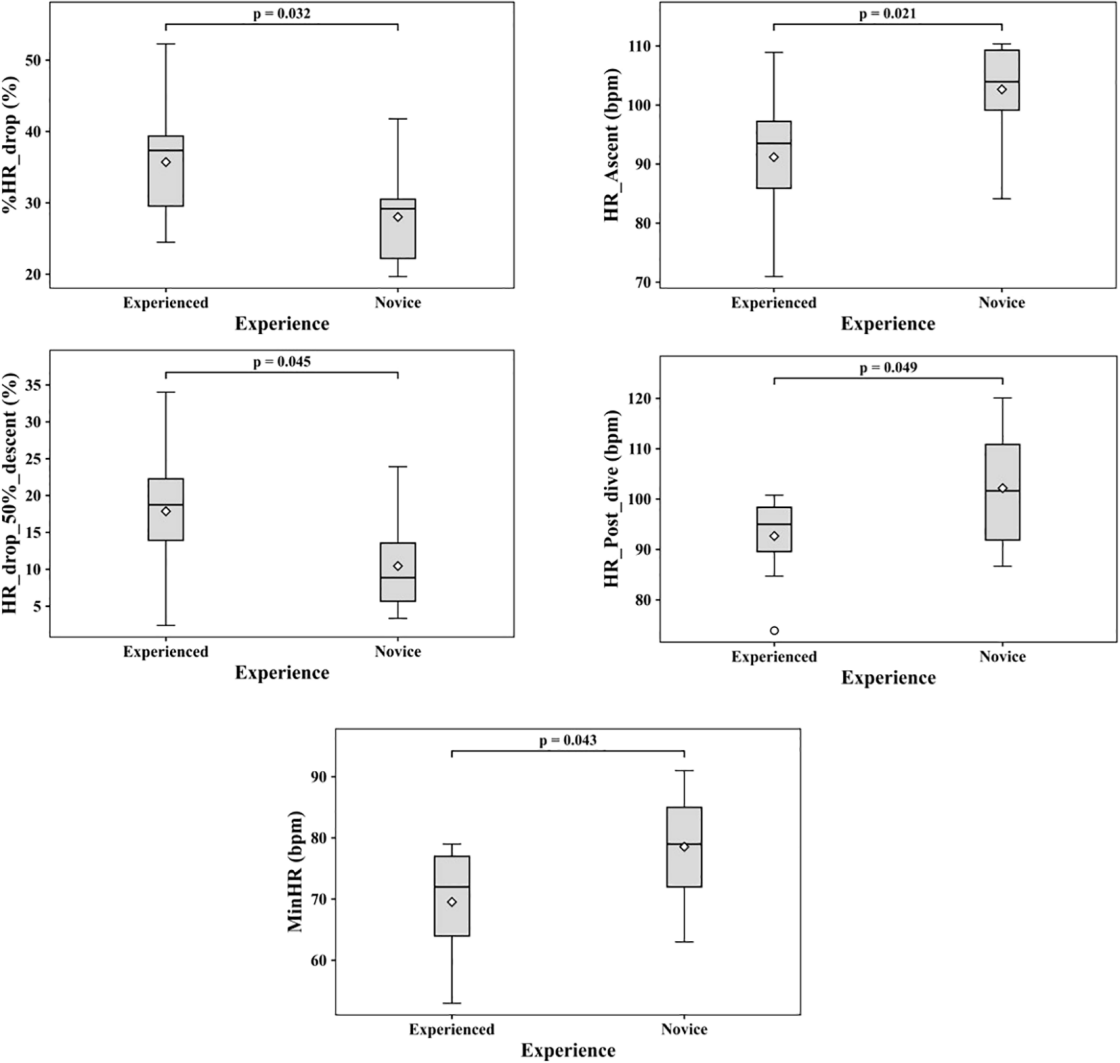
The parameters with uncorrected p-values< 0.05 between experienced and novice divers. No comparison remained significant after BH-FDR correction, and all comparisons shown here are therefore exploratory.

The cardiovascular reflex indices for experienced and novice divers are presented in **Table 4**. This analysis is conducted to provide descriptive context for group differences in phase-wise heart rate and derived cardiovascular reflex indices. The comparative analysis between novice and experienced divers reveals differences in heart rate across phases and in cardiovascular reflex indices. The %HR_drop is higher among experienced divers compared to novices. The heart rate for all phases is higher for novice divers compared to experienced divers. The %HR_drop, HR_Ascent, HR_post_dive, MinHR, and HR_drop_50%_descent have p-value< 0.05. However, after applying the Benjamini–Hochberg FDR correction, no parameters had q values less than 0.05, indicating that the matrix comparison of **Table 4** should be treated as exploratory. Despite the correction, there are several parameters, MinHR (d=-0.96), %HR_drop (d=1.04), that have a large effect size. HR_bottom and HR_descent have nominal p-values > 0.05, but the indices have large effect sizes (-0.87 and -0.93). A sensitivity analysis was performed using the available sample sizes (Experience: 11 and Novice:9), α = 0.05, and 80% power, and it indicates a minimum detectable effect size of Cohen’s d = 1.33. This corresponds to a minimum detectable difference of approximately 9.79 for %HR_drop and 12.46 bpm for MinHR. The box plot for the features with the uncorrected p-values< 0.05 is presented in **Figure 4**.

**Table 4:** Cardiovascular reflex indices for the experience and novice divers.

Indices	Experience (n=11)	Novice (n=9)	p-value	q-value	Effect Size	CI
HR_rest (bpm)	100.29 ± 11.33	101.27 ± 17.95	0.883^b^	0.884	0.06	-1.02 — 0.96
HR_pre_dive (bpm)	108.33± 10.05	109.72 ± 13.46	0.792^b^	0.849	-0.12	-1.09 — 0.86
HR_descent (bpm)	95.18 ± 8.83	104.95 ± 13.70	0.069^b^	0.148	-0.87	-2.19 — 0.02
HR_bottom (bpm)	82.65 ± 12.74	93.86 ± 10.96	0.054^a^	0.144	-0.93	-1.88 — -0.15
HR_Ascent (bpm)	91.19 ± 11.36	102.64 ± 8.22	0.021^b^	0.144	-1.13	-2.35 — -0.33
HR_post_dive (bpm)	92.67 ± 8.051	102.17 ± 12.06	0.049^b^	0.144	-0.95	-2.09 — -0.13
MinHR (bpm)	69.55 ± 9.77	78.55 ± 8.82	0.043^a^	0.144	-0.96	-1.96 — -0.16
depth_minHR (feet)	74.54 ± 3.37	68.96 ± 16.79	0.62^a^	0.716	0.48	-0.62 — 1.07
Time_minHR (sec)	374 ± 124.52	322.77 ± 147.55	0.410^b^	0.513	0.37	-0.57 — 1.29
Time_minHR_bottom (sec)	245.82 ± 119.19	192.75 ± 78.98	0.289^b^	0.434	0.50	-0.35 — 1.50
Slope_descent (bpm/feet)	-0.36 ± 0.17	-0.23 ± 0.12	0.104^b^	0.174	-0.77	-1.86 — 0.06
Slope_ascent (bpm/feet)	-0.15 ± 0.09	-0.13 ± 0.05	0.361^a^	0.493	-0.09	-0.77 — 1.50
HR_drop (bpm)	38.78 ± 9.57	31.17 ± 10.30	0.104^b^	0.174	0.76	-0.09 — 1.92
%HR_drop (%)	35.73 ± 7.67	28.02 ± 6.93	0.031^b^	0.144	1.04	0.24 — 2.22
HR_drop_50%_descent (%)	17.89 ± 8.57	10.45 ± 6.81	0.045^b^	0.144	0.95	0.12 — 2.33

^a^ Mann-Whitney U ^b^ t-test.

## 4 Discussion

The challenges in SCUBA diving are difficult due to the physiological adaptation of the human body in a high-pressure environment, along with the apparatus, temperature, and underwater physical activity. This challenge resulted in complex phenomena called the diving reflex. The psychological adaptation of divers before diving also plays a crucial role, and it directly affects the safety of the divers. The dives in the present study were conducted in warm water (~28 °C), which likely attenuated the cold-facial component of the trigeminally mediated diving response relative to cold-water conditions. Accordingly, the observed heart-rate changes are specific to the current experimental settings. All participants in the novice group held Dive Master certification, which indicates that the sample did not include truly inexperienced or recreational divers. The present findings reflect differences between relatively lower- and higher-experience certified divers and should not be generalised to non-professional diving populations. The present study examines the changes in heart rate with depth and time during diving in a real-world scenario.

In the pre-dive phase, the divers were instructed to remain in the water with their faces above water and minimal movement. The heart rates of the divers were the highest during this phase. These increases were likely due to the preparatory phase, during which the diver wears the diving suit, sets the dive computer, and checks the gas pressure (Alboni et al., 2011). The heart rate begins to drop during immersion and continues to decrease throughout the descent phase. There is an 8.65% reduction in heart rate from the pre-dive phase to the descent phase. The percentage reduction varies individually and ranges from 5% to 39%, as described in the literature (Schaller et al., 2021). After face immersion, vagal activity increases due to ANS stimulation (Gooden, 1994; Noh et al., 2018). Hydrostatic pressure moves blood from the lower part of the body into the central circulation, contributing to bradycardia, increased stroke volume, and increased cardiac output. The statistical analysis does not show a significant difference in heart rate between the pre-dive and descent phases, which might be due to inter-subject variability.

During the bottom phase, an interesting observation was noted. The divers maintained a constant depth while their heart rate continued to decrease. The bottom_depth_sd was included as the covariate, and it did not have a significant effect on heart rate. This suggests that the observed HR modulation at the bottom was not accounted for by depth-related variability in the present study. The *post hoc* analysis from EMM shows a significant difference between descent and bottom (p< 0.001) and between pre-dive and bottom (p< 0.001). The decrease in heart rate (21.25 bpm) at the bottom was more than twice the decrease during the descent phase (9.39 bpm) compared to the pre-dive phase. The minimum heart rate was observed when the divers reached the bottom and remained there. The average time from reaching the bottom to the minimum heart rate is approximately 3 minutes 53 seconds, which is 77.6% of their time at the bottom. A similar trend has been reported previously (Fattorini et al., 2024), although it was not statistically significant. In this study, the duration of stay was less (5minutes), but the same observation is confirmed with statistical significance. This decrease reflects the continuous expression of the diving reflex at the bottom, compared to the rapid initial reflex seen during descent. In the bottom phase, the divers are under increased ambient pressure and hyperoxic conditions with elevated inspired oxygen partial pressure. The hyperbaric condition continues to shift the blood to the central region. Thus, even at steady depth, the sustained bradycardia is observed. This is consistent with the experiments conducted in a hyperbaric environment in air, which have shown that bradycardia persists even after 30 minutes, but the decrease in heart rate has slowed down as the body continues to adjust to the condition (Lund et al., 2000). The increase in the partial pressure of oxygen and ambient pressure has been reported to induce peripheral vasoconstriction, and this change is detected by baroreceptors, which in turn cause the heart rate to slow down (Gole et al., 2011; Schipke et al., 2022). In a study conducted by Thomson et al (Thomson et al., 2005), the subjects were exposed to a hyperoxic environment for 1 hour, and it resulted in a decrease in heart rate, which persisted during the exposure hour.

The exploratory analysis of derived cardiovascular indices presented in **Table 4** suggests that experienced divers may reveal a stronger phase-specific bradycardic response compared with novices, as reflected by a higher cohen’s d value for several parameters such as MinHR and %HR_drop. The power analysis indicates the observed effect sizes for the %HR_drop (d = 1.04) and MinHR (d = -0.96) fell below the minimum detectable effect size (d=1.33). These indicate that the study was underpowered for the magnitudes of effect observed in the data. HR_descent and HR_bottom have large negative effect sizes but fail to achieve statistical significance. This could be due to the small sample size (n=20), which makes the study underpowered, leading to non-significant results. The overall percentage of heart rate drop and the heart rate drop at 50% of descent are higher in experienced divers compared to beginners. These findings indicate that experience is associated with differences in the magnitude of heart rate responses during the dive. The probable reason could be a combination of psychological stress as well as cardiovascular adaptability. Prior work has shown that autonomic responses to a hyperbaric environment involve physiological adjustments and psychological stress, which influences the SNS activity (Lund et al., 2000; Anegg et al., 2002; Flouris and Scott, 2009).

The interaction phase through the LMM was informative. The changes in slope are negative in descent, depth and ascent phases which shows that more experienced divers (divers with more logged dives) exhibit lower mean heart rate compared with less experienced divers (divers having less logged dives) in these phases. This aligns with the broader expectation that practice may reduce stress activity and improve efficiency during diving. The two diver groups differed significantly in age, and age was included as a covariate in the LMM. In the present dataset, age was not a significant predictor of the heart rate, and its inclusion did not alter the model results. However, given the modest sample size and the overlap between age and diving experience, residual confounding cannot be fully excluded. The reduction in heart rate among experienced divers may reflect the combined influence of age and accumulated diving exposure rather than an independent effect of experience alone. For the beginners, it is found from prior work that bradycardia follows psychological stress, which decreases the overall heart rate drop. In the literature, the decrease in cortisol levels is found to be greater in experienced divers than in novices, suggesting higher stress levels in novices during diving (Chen et al., 2025). From a physiological perspective, one possibility is that the faster heart rate reduction observed in experienced divers may be influenced by experience-related differences in vascular regulation and potentially better baroreceptor responses. This may result in faster vagal activation and, consequently, a faster decrease in heart rate. In literature, high mean arterial pressure (MAP) resulting from vasoconstriction was observed in elite divers compared to novices in breath-hold diving (Bourdas and Geladas, 2024).

Interestingly, the HR_ascent and HR_post_dive of novice divers is higher than experienced divers. The high effect size of -1.13 and -0.95 for HR_ascent and HR_post dive suggests potential divergence in cardiovascular response between the experienced and novice groups. This elevation may reflect differences in recovery dynamics during ascent. During decompression, there is a shift from parasympathetic activity to sympathetic activity, which causes an increase in heart rate (Chouchou et al., 2009). One more explanation could be that novices had to pay more attention to buoyancy control, ascent rate regulation, gas monitoring, and safety stop, which overall increases the sympathetic activation and increases heart rate. The higher cortisol levels in novices, as reported in previous literature, support the existence of stress (Morgan, 1995; Coetzee, 2011). Experienced individuals may easily handle these adjustments more efficiently and may possess superior recovery mechanisms that meet the physiological and psychological demands of SCUBA diving. Previous literature has reported that repeated exposure to stressors enhances vagal activation and stress handling capacity (Hisamoto et al., 2025; Hu et al., 2025). The adaptive and progressive training can help novice divers to get familiar with the changes in a hyperbaric environment. Relaxation techniques, such as deep breathing and meditation, can help the divers manage stress better. There is an increase in heart rate from the rest phase to the pre-dive phase prior to submersion. Incorporating the stress management technique and regular practice of pre-dive arrangement of divers can help them to deal with the increase in heart rate. Even though greater experience is associated with lower heart rate, it does not eliminate the cardiovascular risk given the autonomic demands of repeated diving exposure. This study collectively emphasises the role of experience in shaping phase-dependent heart rate responses during SCUBA diving and the need for training programs and continuous health monitoring to ensure the safety of divers. Although the present discussion has focused primarily on cardiovascular and stress-related responses, the diving response may engage a broader autonomic-emotional network. Emerging work has suggested that non-cardiac markers, including pupillometric changes (Rizzuto et al., 2025b) during face immersion apnea, may also reflect aspects of this coordinated response. The interpretation of pupillary dynamics as autonomic biomarkers is supported by a wider literature on spontaneous pupillary oscillations, pupillary unrest, and hippus, including their spectral and complexity properties in physiological and clinical contexts (Schumann et al., 2015; Turnbull et al., 2017; Schumann et al., 2020; Rizzuto et al., 2025a). However, such evidence is not directly equivalent to open-water SCUBA diving.

## 5 Limitations of the study

The study had a few limitations. Firstly, the number of participants in this study is limited. However, a small number of participants is very common in similar studies conducted in extreme environments. Secondly, the most experienced participants in this study had up to 12 years of diving experience. Future studies may incorporate a broader range of experiences and a wider age distribution to strengthen generalisability. Thirdly, the duration spent at the bottom of the dive is 5 minutes, which might be insufficient to characterise the stabilisation and cardiovascular adaptation at depth. Future protocols should include an extended time period at the bottom to gain additional insight into experience-dependent adaptability in hyperbaric and hyperoxic environments. The stress is not directly measured in this study, and the difference in vasoconstriction between experienced and novice divers could be studied separately. The phase-specific HRV metrics would strengthen the autonomic interpretation if reliable RR interval data were available. However, HRV could not be computed in the present dataset because the recordings were captured using the Polar H10 built-in memory and subsequently processed via Polar Flow, which does not provide a downloadable HRV/RR-interval file for sessions recorded to H10 internal memory. Polar explicitly notes that only sessions recorded with Polar Watch and H10 will be available in the flow web service. This study primarily assessed diving experience through logged dives and years of experience, although experience is likely to be multidimensional. Psychological factors, including anxiety regulation, interoceptive awareness, stress tolerance, and cognitive strategies, may influence autonomic cardiovascular responses and may differ among divers with comparable exposure histories (Giuliano et al., 2026). Since the current groups varied in age and certification level, the detected heart-rate patterns cannot be solely attached to experience. Subsequent research should implement a more comprehensive, multidimensional operationalisation of diving expertise that encompasses physiological, technical, and psychological aspects.

## 6 Conclusion

The study looks into the cardiovascular response to self-contained underwater breathing apparatus (SCUBA) diving by evaluating heart rate across six phases in divers. The study showed a reduction in heart rate during descent and at the bottom, which is consistent with the bradycardic response. The heart rate continued to decrease, even at a consistent depth at the bottom, due to cardiovascular adaptation under hyperoxic and hyperbaric conditions. The comparative analysis between novice and experienced divers suggested that experienced divers show a stronger bradycardic response during descent and the bottom phase than novice divers. However, because none of the between-group cardiovascular comparisons remained significant after BH-FDR correction, these findings should be treated as exploratory. Novice divers showed a lower percentage drop in heart rate than experienced divers, although this difference should be interpreted as exploratory. The results motivate the need for a customised training program for novice divers to increase adaptability in a hyperbaric environment. The experienced divers should also be regularly screened for cardiovascular disease to avoid any adverse events. The phase-wise monitoring of physiological parameters and the integration of wearable analytics can help to raise an alert in case of an emergency, thus improving the safety of the divers.

## Data Availability

The raw data supporting the conclusions of this article will be made available by the authors, without undue reservation.
