# Correction: Influence of experience on cardiovascular diving reflex in professional divers

**DOI:** 10.3389/fphys.2026.1931513

**Published:** 2026-07-20

**Authors:** Subhojit Jash, Sajan Kapil, S Kanagaraj

**Affiliations:** 1 Center for Intelligent Cyber-Physical Systems, Indian Institute of Technology Guwahati, Assam, India; 2 Department of Mechanical Engineering, Indian Institute of Technology Guwahati, Assam, India; 3 Jyoti and Bhupat Mehta School of Health Sciences and Technology, Indian Institute of Technology Guwahati, Assam, India

**Keywords:** autonomic nervous system, bradycardia, diving reflex, heart rate, SCUBA

Author “S Kanagaraj” was erroneously omitted as a corresponding author. The correct corresponding authors are: “Subhojit Jash, subhojit.jash@iitg.ac.in and S. Kanagaraj kanagaraj@iitg.ac.in”.

The original version of this article has been updated.

