# Supplementary material for: Influence of experience on cardiovascular diving reflex in professional divers
Source: Front Physiol. 2026 Apr 27;17:1793755. doi: 10.3389/fphys.2026.1793755 (PMC13158098; doi:10.3389/fphys.2026.1793755)
Supplement: Supplementary file 1 [file Table1.docx]

Sensitivity Analysis for (a)year-based grouping (b)years of diving as a continuous variable (c) logged dives

**(a)Model: Phase × Experience**

Table S1 LMM analysis of phase * Experience on mean heart rate across diving stages in experienced and novice divers

| **Fixed effect** | **Coefficients** | **p-value** | **CI [2.5-97.5]** | |
| --- | --- | --- | --- | --- |
| **Intercept** | 108.955 | p<0.005 | 103.748 | 114.163 |
| **Group Contrast (Experience, Pre-dive vs. Novice, Pre-dive)** | 1.39 | 0.791 | -8.92 | 11.72 |
| **Phase Contrast** |  |  |  |  |
| Pre-dive vs. Rest | -8.218 | p=0.006 | -12.384 | -4.053 |
| Pre-dive vs. Descent | -9.795 | p<0.005 | -13.960 | -5.629 |
| Pre-dive vs. Bottom | -21.217 | p<0.005 | -25.382 | -17.051 |
| Pre-dive vs. Ascent | -12.649 | p<0.005 | -16.815 | -8.484 |
| Pre-dive vs. Post-dive | -12.012 | p<0.005 | -16.178 | -7.847 |
| **Interaction Contrast** |  |  |  |  |
| Experience vs. Novice, Pre-dive – Rest | -0.42 | p=0.922 | -8.95 | 8.101 |
| Experience vs. Novice, Pre-dive - Descent | 8.38 | p=0.054 | -0.148 | 16.91 |
| Experience vs. Novice, Pre-dive - Bottom | 9.82 | p=0.024 | 1.291 | 18.34 |
| Experience vs. Novice, Pre-dive - Ascent | 10.05 | p=0.021 | 1.52 | 18.57 |
| Experience vs. Novice, Pre-dive - Post-dive | 8.102 | p=0.063 | -0.426 | 16.63 |

**(b)Model: Phase × Years (Continuous) +Age**

Table S2 LMM analysis of phase * Years (Continuous) on mean heart rate across diving stages

| **Fixed effect** | **Coefficients** | **p-value** | **CI [2.5-97.5]** | |
| --- | --- | --- | --- | --- |
| **Intercept** | 108.955 | p<0.005 | 103.748 | 114.163 |
| **Main effect (Slope of Years on HR in Pre-dive phase)** | -0.425 | 0.652 | -2.366 | 3.782 |
| **Phase Contrast** |  |  |  |  |
| Pre-dive vs. Rest | -8.218 | p<0.005 | -12.384 | -4.053 |
| Pre-dive vs. Descent | -9.795 | p<0.005 | -13.960 | -5.629 |
| Pre-dive vs. Bottom | -21.217 | p<0.005 | -25.382 | -17.051 |
| Pre-dive vs. Ascent | -12.649 | p<0.005 | -16.815 | -8.484 |
| Pre-dive vs. Post-dive | -12.012 | p<0.005 | -16.178 | -7.847 |
| **Interaction Contrast** |  |  |  |  |
| Pre-dive - Rest (changes in slopes) | 0.130 | p=0.828 | -1.048 | 1.309 |
| Pre-dive – Descent (changes in slopes) | -1.111 | p=0.065 | -2.289 | 0.067 |
| Pre-dive – Bottom (changes in slopes) | -1.126 | p=0.061 | -2.305 | 0.052 |
| Pre-dive – Ascent (changes in slopes) | -0.765 | p=0.203 | -1.943 | 0.414 |
| Pre-dive - Post-dive (changes in slopes) | -0.631 | p=0.294 | -1.810 | 0.547 |
| **Age (Covariate)** | -0.699 | p=0.416 | -3.407 | 1.410 |

**(c) Model: Phase × logged dives + Age**

Table S3 LMM analysis of phase * logged dives with age as covariate on mean heart rate across diving stages

| **Fixed effect** | **Coefficients** | **p-value** | **CI [2.5-97.5]** | |
| --- | --- | --- | --- | --- |
| **Intercept** | 108.955 | p<0.005 | 103.748 | 114.163 |
| **Main effect (Slope of logged dives on HR in Pre-dive phase)** | 5.273 | 0.724 | -16.645 | 23.956 |
| **Phase Contrast** |  |  |  |  |
| Pre-dive vs. Rest | -8.218 | p<0.005 | -12.384 | -4.053 |
| Pre-dive vs. Descent | -9.795 | p<0.005 | -13.960 | -5.629 |
| Pre-dive vs. Bottom | -21.217 | p<0.005 | -25.382 | -17.051 |
| Pre-dive vs. Ascent | -12.649 | p<0.005 | -16.815 | -8.484 |
| Pre-dive vs. Post-dive | -12.012 | p<0.005 | -16.178 | -7.847 |
| **Interaction Contrast** |  |  |  |  |
| Pre-dive - Rest (changes in slopes) | 1.411 | p=0.821 | -10.842 | 13.665 |
| Pre-dive – Descent (changes in slopes) | -14.367 | p=0.022 | -26.621 | -2.114 |
| Pre-dive – Bottom (changes in slopes) | -15.568 | p=0.013 | -27.821 | -3.314 |
| Pre-dive – Ascent (changes in slopes) | -17.429 | p=0.005 | -29.683 | -5.176 |
| Pre-dive - Post-dive (changes in slopes) | -10.261 | p=0.101 | -22.514 | 1.993 |
| **Age (Covariate)** | -0.694 | p=0.307 | -2.026 | 0.638 |

Sensitivity Analysis for Depth:

**Model: Phase × logged dives + Age + bottom_depth_sd**

Table S4 LMM analysis of phase * logged dives with age as covariate on mean heart rate across diving stages

| **Fixed effect** | **Coefficients** | **p-value** | **CI [2.5-97.5]** | |
| --- | --- | --- | --- | --- |
| **Intercept** | 108.955 | p<0.005 | 103.748 | 114.163 |
| **Main effect (Slope of logged dives on HR in Pre-dive phase)** | 5.273 | 0.724 | -16.645 | 23.956 |
| **Phase Contrast** |  |  |  |  |
| Pre-dive vs. Rest | -8.218 | p<0.005 | -12.384 | -4.053 |
| Pre-dive vs. Descent | -9.795 | p<0.005 | -13.960 | -5.629 |
| Pre-dive vs. Bottom | -21.217 | p<0.005 | -25.382 | -17.051 |
| Pre-dive vs. Ascent | -12.649 | p<0.005 | -16.815 | -8.484 |
| Pre-dive vs. Post-dive | -12.012 | p<0.005 | -16.178 | -7.847 |
| **Interaction Contrast** |  |  |  |  |
| Pre-dive - Rest (changes in slopes) | 1.411 | p=0.821 | -10.842 | 13.665 |
| Pre-dive – Descent (changes in slopes) | -14.367 | p=0.022 | -26.621 | -2.114 |
| Pre-dive – Bottom (changes in slopes) | -15.568 | p=0.013 | -27.821 | -3.314 |
| Pre-dive – Ascent (changes in slopes) | -17.429 | p=0.005 | -29.683 | -5.176 |
| Pre-dive - Post-dive (changes in slopes) | -10.261 | p=0.101 | -22.514 | 1.993 |
| **Age (Covariate)** | -0.694 | p=0.307 | -2.026 | 0.638 |
| **bottom_depth_sd** | 6.875 | p=0.476 | -12.035 | 25.784 |

Sensitivity Analysis for Baseline

**Model: Phase × logged dives + Age (Rest as Reference)**

Table S5 LMM analysis of phase * logged dives with age as covariate on mean heart rate across diving stages and with Rest as reference

| **Fixed effect** | **Coefficients** | **p-value** | **CI [2.5-97.5]** | |
| --- | --- | --- | --- | --- |
| **Intercept** | 100.737 | p<0.005 | 95.615 | 105.859 |
| **Main effect (Slope of logged dives on HR in Rest phase)** | 6.684 | 0.724 | -12.881 | 26.248 |
| **Phase Contrast** |  |  |  |  |
| Rest vs. Pre-dive | 8.218 | p<0.005 | 4.053 | 12.384 |
| Rest vs. Descent | -1.576 | p<=0.458 | -5.742 | 2.589 |
| Rest vs. Bottom | -12.998 | p<0.005 | -17.164 | -8.832 |
| Rest vs. Ascent | -4.431 | p<0.037 | -8.596 | -0.265 |
| Rest vs. Post-dive | -3.794 | p<0.074 | -7.960 | 0.372 |
| **Interaction Contrast** |  |  |  |  |
| Rest - Pre-dive (changes in slopes) | -1.411 | p=0.821 | -13.665 | 10.842 |
| Rest – Descent (changes in slopes) | -15.779 | p=0.012 | -28.032 | -3.525 |
| Rest – Bottom (changes in slopes) | -16.979 | p=0.007 | -29.232 | -4.725 |
| Rest – Ascent (changes in slopes) | -18.840 | p=0.003 | -31.094 | -6.587 |
| Rest - Post-dive (changes in slopes) | -11.672 | p=0.062 | -23.925 | 0.582 |
| **Age (Covariate)** | -0.694 | p=0.307 | -2.026 | 0.638 |
